# Changes of Body Mass Index and Body Shape in relation to risk of Gastric Cancer: A population-based case-control study

**DOI:** 10.7150/jca.56149

**Published:** 2021-03-23

**Authors:** Xiaolin Yin, Xiaorong Yang, Tongchao Zhang, Ziyu Yuan, Hui Chen, Li Jin, Xingdong Chen, Ming Lu, Weimin Ye

**Affiliations:** 1 Department of Epidemiology and Biostatistics, School of Public Health, Cheeloo College of Medicine, Shandong University, Jinan, China.; 2 Clinical Epidemiology Unit, Qilu Hospital of Shandong University, Jinan, China.; 3 Clinical Research Center of Shandong University, Jinan, China.; 4 State Key Laboratory of Genetic Engineering, Collaborative Innovation Center for Genetics and Development, School of Life Sciences, Fudan University, Shanghai, China.; 5 Fudan University Taizhou Institute of Health Sciences, Taizhou, China.; 6 Human Phenome Institute, Fudan University, Shanghai, China.; 7 Department of Medical Epidemiology and Biostatistics, Karolinska Institutet, Stockholm, Sweden.; 8 Department of Epidemiology and Health Statistics & Key Laboratory of Ministry of Education for Gastrointestinal Cancer, Fujian Medical University, Fuzhou, China.

**Keywords:** body mass index, body shape, gastric cancer, *Helicobacter pylori*, case-control study

## Abstract

**Background:** The results of previous studies are heterogeneous about the effect of body fatness on risk of gastric cancer (GC). Herein we investigated the effect of changes of BMI and body shape on risk of GC.

**Methods:** A population-based case-control study enrolled 1989 controls and 937 GC cases. Logistic regression models were used to calculate odd ratios (*OR*s) and 95% confidence intervals (*CI*s) for BMI and body shape in association with GC risk, according to anatomical subsite, Laurén's classification, sex and *Helicobacter pylori* (*Hp*) infection.

**Results:** Subjects with higher BMI or body shape 10 years before interview had a lower risk of GC regardless of anatomical subsite, Laurén's classification, and sex (all *P* for trend <0.05). But the relative risk patterns were different by *Hp* status. When checking the effect of changes of body fatness, in *Hp*+ stratum, the *OR*s (95% *CI*) were 0.40 (0.17-0.93) for subjects who were underweight at age 20 but had increased BMI afterwards, and 0.48 (0.32-0.73) for those of body shape 1/2 at age 20 but increased body shape subsequently, compared to subjects with stable BMI or body shape. When subjects had a normal BMI or 3/4 body shape at age 20, weight loss nearly doubled the risk of GC, and weight gain would decrease the risk.

**Conclusion:** The association between body fatness and GC risk might differ by time point of measurement and *Hp*-infection status. Further, the influence of changes of body fatness might be different by baseline body fatness and *Hp*-infection status.

## Introduction

Gastric cancer (GC) is one of the most dreadful malignancy around the world, with over 1,000,000 new cases and an estimated 783,000 deaths reported in 2018 1. China is a high-risk area for GC, and there were nearly 44% of GC cases and 50% of the deaths from GC occurring in China alone 2. Besides *Helicobacter pylori* (*Hp*) infection 3, other well-established risk factors include smoking 4, high dietary salt intake 5, and family history of GC or gastric mucosal abnormality 6. Nevertheless, it remains in dispute whether body fatness is associated with the risk of GC. Prevalence of overweight and obesity has increased rapidly in China, especially among children and adolescents 7,8. Overweight and obesity has become a major health challenge 7, and more attention is needed to explore its association with the risk of GC.

GC can be subdivided by anatomic subsite into esophagogastric-junction cancer (EGJC) and true gastric cancer (TGC, cancer in the other areas of the stomach) 9, and they might be differently influenced by overweight and obesity. Some studies reported significant positive association between overweight or obesity and the risk of EGJC 10-13, while some did not 14. On the other hand, Levi *et al* found that adolescent obesity was associated with an increased risk of subsequent TGC in both men and women 15. However, Fan *et al* pointed out that higher body mass index (BMI) was associated with a decreased risk of TGC, particularly in men and older persons 16. Besides, Laurén divided GC into intestinal type and diffuse type, according to the presence of glandular growth pattern 17, and the two histopathological subtypes reportedly show different trends in incidence or etiology 18.

BMI is one of the most used measures to assess body fatness, and it has high specificity but low sensitivity to identify adiposity 19. In addition, assessment of body shape was also used as an indicator for body fatness, the validity of which was verified in the Boston-based Third Harvard Growth Study 20. Moreover, although there were many studies considering the changes in body fatness, they didn't take into account of the effect of baseline body fatness 11,21.

Taixing of Jiangsu Province, situating in Eastern China, is a high incidence area of GC 22. A population-based case-control study of upper gastrointestinal cancer was conducted in this area since 2009. The present study aims to examine the associations between changes in BMI and body shape and risk of GC.

## Methods

### Study population

The detailed design of this population-based case-control study could be found in our previous publications 23,24. In brief, we carried out a population-based case-control study in Taixing of Jiangsu province, from October 2010 to September 2013.

### Recruitment of cases and controls

**Figure 1:**
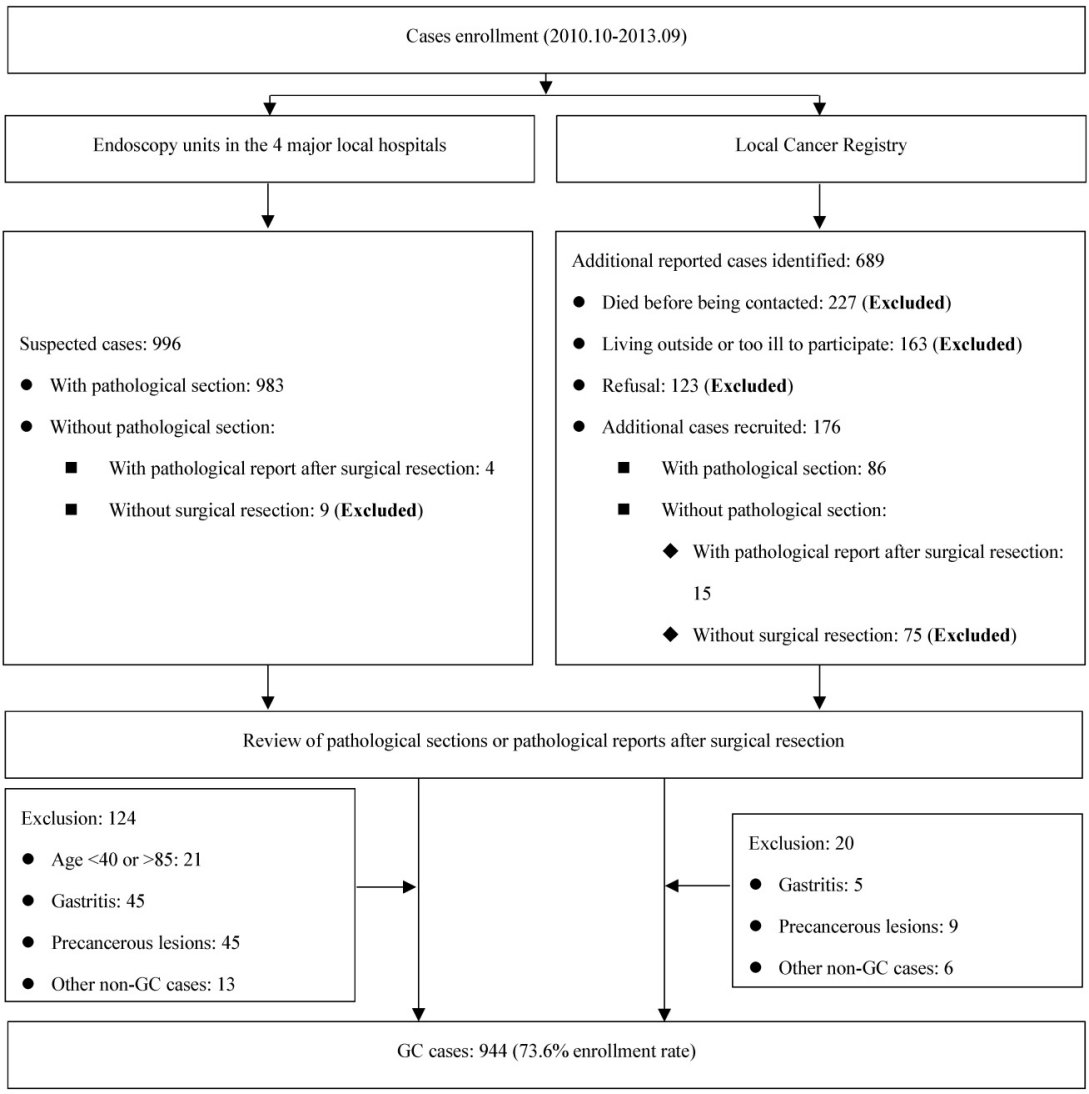
Flow diagram of inclusion/exclusion criteria for cases.

Eligible participants were limited to those between ages of 40 and 85 years, and living in Taixing for at least 5 years. GC cases were recruited if the patients were suspected as GC cases during gastroscopy examination in four largest local hospitals (the Taixing People's Hospital, the Second People's Hospital of Taixing, the Taixing Chinese Medicine Hospital and the Third People's Hospital of Taixing), where more than 90% of the patients in Taixing were diagnosed. And we established a rapid case recruitment system that directly notified our study staff to interview the suspected GC cases. To find all potential GC cases, a cross-matching with the local Cancer Registry's list of cases was made each year to identify any missing cases. Finally, all cases were examined by a pathologist and excluded if not confirmed. The detailed process is shown in Figure 1.

Controls were from the local Population Registry System, which covers the whole population of Taixing. They were randomly selected every 12 months and a frequency matching method for control selection was used to increase statistical power. We recruited the controls at the same time for all upper gastrointestinal cancer cases because of the approximately identical distribution of age and sex. All upper gastrointestinal cancer cases were stratified by sex and 5-years age groups, and corresponding controls were selected by an appropriate ratio for each stratum, considering the response rate among controls. In this way, 3501 controls were randomly selected, of whom 643 were excluded because of the death before being contacted, outmigration or inability to be contacted. Finally, 2011 controls participated in this study (response rate: 70.4%), of whom 19 controls were out of the age range (40-85 years) and 1992 controls were included in the final dataset.

### Data collection

All participants were interviewed by trained interviewers face to face, using an electronic questionnaire. Cases were under investigation before they knew their diagnoses of cancer. Data on demographic characteristics and other study variables were obtained, including the body weight, height and body shape at age 20 and 10 years before interview. BMI was calculated as the body weight in kilograms divided by the square of the height in meters and divided into four categories: underweight, < 18.5 kg/m^2^; normal, ≥ 18.5 to < 24 kg/m^2^; overweight, ≥ 24 to < 28 kg/m^2^; obesity, ≥ 28 kg/m^2^. Body shape was chosen from the revised image of somatotypes (Figure S1) 25, from which we deleted the two highest levels for men considering the local prevalence of obesity. For women, due to small numbers, those with body shape > 7 were included in the body shape 7 group. Family wealth score, classified according to quintiles, was created based on the ownership of specific household appliances and other variables. Family history of GC was defined as having at least one GC reported in first-degree relatives. Job intensity was classified into four categories based on physical labor (1 for which is a sedentary job, 2 for which is easy to finish with little effort, 3 for which can increase heart rate and sweating slightly, and 4 for which is hard to finish with heart rate increasing significantly and sweating a lot). Meanwhile, serum samples collected from controls and cases were used to detect *Hp* infection by immunoblot assay (Syno Gene Digital Technology, Taizhou, China). In this study, *Hp* infection was defined as *Yes* if the Ure-B antibody was seropositive (cut-off value ≥ 5).

### Subtypes by anatomical subsite, and Laurén's classification

Anatomically, GC cases were divided into EGJC and TGC based on the illustration of tumor sites in their gastroscopy reports. Meanwhile, GC cases were also classified as intestinal type and diffuse type, according to Laurén's classification 17.

### Statistical analysis

Distribution of demographic characteristics and other study variables was presented as mean and standard deviation for continuous variables, and frequency and percentage for categorical variables. Unconditional logistic regression models were used to calculate odds ratios (*OR*s) with 95% confidence intervals (*CI*s) for BMI and body shape in association with risk of GC, according to anatomical classification, Laurén's classification, sex or *Hp* infection, respectively. All relative risk estimates were adjusted for the following potential confounding factors: age (continuous), sex (male or female), education (illiteracy, primary school, junior school or high school and above), marital status (unmarried, married or divorced/widowed), occupation (farmer, worker or others), *Hp* infection (yes or no), sum of missing and filled teeth (none, < 6 or ≥ 6), daily frequency of brushing teeth (< 2 or ≥ 2), tea drinking (yes or no), smoking (never, ≤ 30 or > 30 pack-years), alcohol drinking (never, ≤ 80 or > 80 g/day), job intensity (1, 2, 3 or 4), family wealth score (quintiles) and family history of GC among first-degree relatives (yes or no). Likelihood ratio tests were used to test the effect modification by family wealth, smoking, alcohol drinking and job intensity. Then, sensitivity analyses were undertaken by excluding cases only from the local Cancer Registry and by excluding subjects older than 75. All analyses were performed using Stata software (version 14.0). All tests were two-sided and *P*-values less than 0.05 were considered significant.

### Ethics approval

The study protocol was approved by the Institutional Review Board of the School of Life Sciences, Fudan University and the Institutional Review Board of Qilu Hospital, Shandong University. Written informed consent was received from all participants.

## Results

The study was based on 1989 controls and 937 cases, after excluding 10 subjects with incomplete information on our concerned variables.

Table 1 presents the demographic characteristics of all participants. The mean age was slightly higher in cases than controls. The distribution of sex showed no significant difference. Compared with controls, cases had lower educational level, and were less likely to be married, more likely to have *Hp* infection. Further, they had higher sum of missing and filled teeth, less daily frequency of brushing teeth, more daily alcohol drinking, higher-intensity work, and were more likely to have family history of GC among first-degree relatives.

The associations between BMI and body shape in different time points (age 20 and 10 years before interview) and risks of GC are shown in Table 2. BMI or body shape at age 20 did not have significant effect on risk of GC. However, compared with participants with normal BMI 10 years before interview, those with lower BMI had an increased risk of GC, while those with higher BMI had a decreased risk of GC (*P* for trend < 0.001). This pattern also applied to body shape 10 years before interview, and a more obvious relative risk gradient was observed. Analysis by anatomic subsite showed similar results (Table S1), and so did when stratified by Laurén's classification (Table S2) and sex (Table S3).

Table 3 presents results of a stratified analysis by *Hp* infection. In the *Hp+* stratum, compared with those with normal BMI 10 years before interview, underweight subjects had an increased risk of GC, while overweight or obese subjects had a lower risk of GC (*P* for trend < 0.001). The relative risk pattern was similar for body shape 10 years before interview (compared with body shape 3, *OR* for those with body shape 1 was 3.25, while for those with body shape 6 or 7, *OR* was 0.57; *P* for trend < 0.001). No significant associations were found between BMI or body shape at age 20 and the risk of GC. However, in the *Hp*- stratum, relative risk patterns were different. Although there was no clear trend of ORs in association with BMI, higher body shape at age 20 was associated with an increased risk of GC (*P* for trend = 0.018). Compared with those with body shape 3 10 years before interview, *OR*s was 2.62 (95% *CI*, 1.10-6.25) for body shape 1, and 2.12 (95% *CI*, 1.26-3.59) for body shape 5.

We further explored the effect of change in BMI and body shape stratified by *Hp* infection (Table 4). In the *Hp+* stratum, when subjects were underweight at age 20, those with normal, overweight or obese BMI 10 years before interview had a 60% decreased risk of GC (*OR*=0.40; 95% *CI*, 0.17-0.93) compared with those keeping underweight all the time. Compared with those who had a normal BMI both at age 20 and 10 years before interview, those with decreased BMI 10 years before interview had an almost doubled risk of GC (*OR*=1.94; 95% *CI*, 1.13-3.31), while those with increased BMI had a 41% decreased risk of GC (*OR*=0.59; 95% *CI*, 0.44-0.80). For change in body shape, the relative risk patterns were almost similar. Compared with those with stable body shape 1/2, those with body shape 1/2 at age 20 but higher body shape 10 years before interview had a more than 50% decreased risk of GC (*OR*=0.48; 95% *CI*, 0.32-0.73). And compared with those with stable body shape 3/4, those with lower body shape later had an almost doubled risk of GC (*OR*=1.96; 95% *CI*, 1.30-2.94). In the *Hp-* stratum, the direction of most point estimates was consistent, although almost all results were not statistically significant, except that those with lower body shape later had an increased risk of GC (*OR*=2.01; 95% *CI*, 1.06-3.82) compared with those with stable body shape 3/4 from age 20.

Besides, analyses of possible effect modification by family wealth, smoking, alcohol drinking, and job intensity were performed. The results were consistent and likelihood ratio tests did not detect any statistically significant interaction (data not shown; all *p*-value of interaction >0.05). Finally, sensitivity analysis was respectively performed by excluding 60 male cases and 21 female cases collected only from the local Cancer Registry, and by excluding subjects older than 75. The results did not change materially (data not shown).

## Discussion

In this large population-based case-control study, we examined the associations between body fatness and risk of GC. Higher BMI and body shape 10 years before interview was negatively associated with risk of GC, regardless of anatomical subsite, Laurén's classification, and sex. And it was found that higher BMI and body shape 10 years before interview was a significant protective factor of GC in the *Hp+* stratum, while in the *Hp*- stratum, higher body shape at age 20 was positively associated with the risk of GC. Furthermore, weight gain after age 20 could decrease risk of GC, while weight loss could increase the risk for normal-weight or underweight persons, although this association was not statistically significant in the *Hp*- stratum.

It remained controversial for the association between body fatness and risk of GC, when GC was separated into EGJC and TGC by anatomical subsite 9-16,26-28. A recent meta-analysis with 24 prospective studies found that higher BMI is positively associated with the risk of EGJC 26. In our study, however, it was found that higher BMI 10 years before interview was negatively associated with risk of EGJC. A cohort study from Linxian of China reported that higher BMI was associated with a decreased risk of TGC (hazard ratio, 0.65; 95% *CI*, 0.51-0.83) 16, as was also found in our study. A population-based cohort study of 5-24 million UK adults reported similar results, that the lower BMI group (BMI < 22 kg/m^2^) had a higher risk of overall GC 29. Only a few studies explored the effect of body fatness at age 20 11,30. Merry *et al* found a higher risk of EGJC with increasing BMI at age 20 11, while Wu *et al* found the same trends for TGC 30. However, we found no significant association between BMI at age 20 and these two subtypes.

Meanwhile, we explored two other subtypes, intestinal type and diffuse type, according to Laurén's classification 17. These two subtypes had some biological differences 17, and the epidemiological and clinicopathological characteristics tend to be different 18. However, we found that the effects of body fatness showed similar patterns on these two subtypes.

*Hp* was classified as a Group I carcinogen for GC by the International Agency for Research on Cancer (IARC) 3, and we conducted a stratified analysis by *Hp* infection. The protective effect of higher BMI 10 years before interview was just found in the *Hp+* stratum. A possible explanation was that weight loss could be caused by *Hp* infection. Lender *et al* found a negative association between *Hp* infection and BMI level 31. *Hp* infection could cause a series of gastric diseases, such as chronic gastritis 32, and then lead to GC eventually 33. When infected with *Hp*, subjects might suffer from chronic gastritis and other gastric diseases for a long time, even more than 10 years, which could cause their weight loss by affecting food intake or digestion.

In our study, among subjects with *Hp* infection, those underweight or with normal-BMI (BMI < 24 kg/m^2^) at age 20 had a higher risk of GC in association with a decreased BMI 10 years before interview, and a lower risk with gain in BMI. There were few studies considering the effects of changes in body fatness 11,21. Audrey *et al* calculated as BMI at baseline minus BMI at age 20 years and found that subjects with a BMI gain of >8.0 kg/m^2^ had a 2.07 times higher risk (95% *CI*, 1.08-3.97) of EGJC compared to subjects with 0-3.9 kg/m^2^ change in BMI 11, while Petrick *et al* only found modestly association of weight change with EGJC 21. Nevertheless, the same BMI change might have different impacts with different baseline BMI. Subjects in our study were divided into 3 groups by baseline BMI, and we found the effects were differential by baseline BMI.

There were other commonly used measurements of body fatness besides BMI, such as abdominal diameter 34, waist circumference and waist-to-hip ratio 12. In order to control for recall bias, we finally chose body shape as another measurement of body fatness, considering that the majority of participants had low educational level and might have a better photographic memory about their body shape. Participants were asked to choose one of the nine pictorial body shape for women (7 for men, modified as few men had a body shape > 7 there) developed by Stunkard *et al* to depict their body shape 25. Although the relative risk patterns were almost similar for body shape, there were some different results in the *Hp*- stratum. It was found that higher body shape at age 20 was positively associated with risk of GC, which was consistent with the findings of Merry *et al* and Wu *et al* 11,30. Besides, a cohort study found that there was a J-shaped association between body fatness (measured by BMI) and GC 35, and we found that subjects with body shape 1 or 5 10 years before interview had a higher risk of GC, when subjects with body shape 3 were chosen as reference.

Discrepancies between our results with those of previous studies might be due to different study populations. Several studies in Asian countries showed a borderline significant or no associations between BMI and risk of GC 27,36-38. A meta-analysis of cohort studies conducted a subgroup analysis on the basis of race, and there was a statistically significant link between excess body weight and GC among non-Asians, but not among Asians 27. Moreover, birth periods of our study population were about 1950s, when a famine plagued nearly all over China. And a study found that Chinese famine then would increase the risk of GC 39.

The biological mechanism is unclear for the association between body fatness and risk of GC. It has been realized that adipose tissue is not only a reservoir for energy storage, but also an essential metabolic and endocrine organ 40,41. Most adipose tissue can express and secrete adipocytokines and other mediators with important immune and endocrine functions, which exert autocrine, paracrine or endocrine effects on neighboring cells, remote tissues and organs 41. Weight loss may impact on body's metabolism and immune function, further to impact on health and even cause cancer. This hypothesis needs to be verified in future studies.

Besides the use of both BMI and body shape to assess body fatness, there are some other strengths in our study. First, we explored the associations for different classifications of GC. Second, our trained staff interviewed cases before they knew their cancer diagnoses, in order to control recall bias. Furthermore, all cases were carefully verified by a pathologist and a high response rate for both cases and controls was also the strength.

Our study also has several limitations. We did not gather information of BMI and body shape at more time points, so that the current results only indicate the association between a relative long-term change and risk of GC. Furthermore, recall bias cannot be absolutely eliminated. But we made efforts to control it in aforementioned ways and found no material change when conducting a sensitivity analysis by excluding cases only from the local Cancer Registry.

In summary, body fatness is associated with GC risk, especially in the time point closer to cancer development. Changes of body fatness also have differential effects on GC risk by baseline body fatness and *Hp* infection status. Further studies are needed to clarify whether the observed effect of body fatness on GC risk is due to the development of precancerous lesions, or on the pathway between *Hp* infection to cancer development, or other to-be-proven mechanisms.

## Supplementary Material

Supplementary figures and tables.

## Figures and Tables

**Table 1:** Demographic characteristics among the study participants

Characteristics	Controls (N=1989)	GC (N=937)	*P*^a^
Age, mean ± SD, years	66.23 ± 8.78	68.10 ± 8.92	**<0.001**
**Sex, n (%)**			
Men	1371 (68.93)	671 (71.61)	0.140
Women	618 (31.07)	266 (28.39)	
**Education, n (%)**			
Illiteracy	538 (27.05)	295 (31.48)	**0.001**
Primary school	759 (38.16)	382 (40.77)	
Junior school	530 (26.65)	191 (20.38)	
High school and above	162 (8.14)	69 (7.36)	
**Marital status, n (%)**			
Unmarried	67 (3.37)	30 (3.20)	**0.009**
Married	1587 (79.79)	705 (75.24)	
Divorced/Widowed	335 (16.84)	202 (21.56)	
**Occupation, n (%)**			
Farmer	1257 (63.20)	632 (67.45)	0.069
Worker	401 (20.16)	173 (18.46)	
Others	331 (16.64)	132 (14.09)	
***Hp* infection**			
No	649 (32.63)	229 (24.44)	**<0.001**
Yes	1319 (66.31)	702 (74.92)	
Missing	21 (1.06)	6 (0.64)	
**Sum of missing and filled teeth, n (%)**			
None	500 (25.14)	195 (20.81)	**0.038**
<6	717 (36.05)	317 (33.83)	
≥6	749 (37.66)	380 (40.55)	
Missing	23 (1.16)	45 (4.80)	
**Daily frequency of brushing teeth, n (%)**			
<2	1290 (64.86)	749 (79.94)	**<0.001**
≥2	677 (34.04)	152 (16.22)	
Missing	22 (1.11)	36 (3.84)	
**Tea drinking, n (%)**			
No	1435 (72.15)	650 (69.37)	0.991
Yes	526 (26.45)	238 (25.40)	
Missing	28 (1.41)	49 (5.23)	
**Smoking, n (%), pack-years**			
Never	884 (44.44)	369 (39.38)	0.215
≤30	542 (27.25)	250 (26.68)	
>30	535 (26.90)	264 (28.18)	
Missing	28 (1.41)	54 (5.76)	
**Alcohol drinking, n (%), g/day**			
Never	1158 (58.22)	479 (51.12)	**0.034**
≤80	404 (20.31)	211 (22.52)	
>80	398 (20.01)	198 (21.13)	
Missing	29 (1.46)	49 (5.23)	
**Job intensity, n (%)**			
1	65 (3.27)	23 (2.45)	**0.009**
2	185 (9.30)	79 (8.43)	
3	300 (15.08)	101 (10.78)	
4	1426 (71.69)	708 (75.56)	
Missing	13 (0.65)	26 (2.77)	
**Family wealth score, n (%)**			
Q1-the lowest	398 (20.01)	204 (21.77)	0.171
Q2	415 (20.86)	219 (23.37)	
Q3	382 (19.21)	171 (18.25)	
Q4	429 (21.57)	199 (21.24)	
Q5-the highest	365 (18.35)	144 (15.37)	
**Family history of gastric cancer among first-degree relatives, n (%)**			
No	1752 (88.08)	727 (77.59)	**<0.001**
Yes	217 (10.91)	170 (18.14)	
Missing	20 (1.01)	40 (4.27)	

Abbreviations: GC, gastric cancer; SD, standard deviation; *Hp, Helicobacter pylori*. Note: Boldface indicates significant difference (*P*-value < 0.05). ^a^ *P*-values were derived from Wilcoxon rank-sum test for continuous variables, and Chi-squared test or Fisher exact test for categorical variables after excluding the corresponding missing values.

**Table 2:** The ORs and 95% CIs for BMI and body shape in association with risk of GC cases

Anthropometric parameters	Controls N (%)	GC N (%)	Unadjusted OR (95% CI)	Adjusted OR (95% CI)^a^
**BMI at age 20**				
Underweight	221 (11.11)	92 (9.82)	0.87 (0.67~1.13)	0.94 (0.71~1.25)
Normal	1343 (67.52)	641 (68.41)	1.00 (reference)	1.00 (reference)
Overweight	375 (18.85)	179 (19.10)	1.00 (0.82~1.22)	1.03 (0.83~1.28)
Obesity	50 (2.51)	25 (2.67)	1.05 (0.64~1.71)	1.09 (0.65~1.85)
*P* for trend			0.455	0.536
**BMI 10 years before interview**				
Underweight	110 (5.53)	93 (9.93)	1.72 (1.28~2.30)	1.53 (1.11~2.11)
Normal	1208 (60.73)	594 (63.39)	1.00 (reference)	1.00 (reference)
Overweight	542 (27.25)	214 (22.84)	0.80 (0.67~0.97)	0.82 (0.67~1.00)
Obesity	129 (6.49)	36 (3.84)	0.57 (0.39~0.83)	0.54 (0.36~0.83)
*P* for trend			**<0.001**	**<0.001**
**Body shape at age 20**				
Shape 1	110 (5.53)	53 (5.66)	1.01 (0.71~1.43)	1.10 (0.76~1.60)
Shape 2	458 (23.03)	202 (21.56)	0.92 (0.75~1.14)	0.92 (0.74~1.16)
Shape 3	744 (37.41)	355 (37.89)	1.00 (reference)	1.00 (reference)
Shape 4	496 (24.94)	230 (24.55)	0.97 (0.79~1.19)	0.97 (0.78~1.21)
Shape 5	143 (7.19)	75 (8.00)	1.10 (0.81~1.49)	1.29 (0.93~1.79)
Shape 6/7	38 (1.91)	22 (2.35)	1.21 (0.71~2.08)	1.31 (0.74~2.33)
*P* for trend			0.378	0.232
**Body shape 10 years before interview**				
Shape 1	50 (2.51)	69 (7.36)	2.97 (2.02~4.37)	3.14 (2.08~4.76)
Shape 2	318 (15.99)	185 (19.74)	1.25 (1.00~1.57)	1.21 (0.95~1.54)
Shape 3	695 (34.94)	323 (34.47)	1.00 (reference)	1.00 (reference)
Shape 4	587 (29.51)	217 (23.16)	0.80 (0.65~0.98)	0.85 (0.69~1.06)
Shape 5	243 (12.22)	119 (12.70)	1.05 (0.82~1.36)	1.19 (0.91~1.57)
Shape 6/7	96 (4.83)	24 (2.56)	0.54 (0.34~0.86)	0.58 (0.35~0.97)
*P* for trend			**<0.001**	**<0.001**

Abbreviations: GC, gastric cancer; BMI, body mass index; ORs, odd ratios; CIs, confidence intervals. Note: Boldface indicates that *P* for trend < 0.05. ^a^ Adjusted for age, sex, education, marital status, occupation, sum of missing and filled teeth, daily frequency of brushing teeth, tea drinking, smoking, alcohol drinking, *Hp* infection, job intensity, family wealth score and family history of GC among first-degree relatives.

**Table 3:** The ORs and 95% CIs for BMI and body shape in association with risk of GC cases, stratified by *Hp* infection

Anthropometric parameters	*Hp* infection (+)				*Hp* infection (-)			
	Controls N (%)	GC N (%)	Unadjusted* OR* (95% CI)	Adjusted* OR* (95% CI)^a^	Controls N (%)	GC N (%)	Unadjusted OR (95% CI)	Adjusted OR (95% CI)^a^
**BMI at age 20**								
Underweight	139 (10.54)	66 (9.40)	0.87 (0.63~1.18)	0.93 (0.67~1.30)	79 (12.17)	25 (10.92)	0.92 (0.57~1.50)	0.95 (0.56~1.61)
Normal	894 (67.78)	490 (69.80)	1.00 (reference)	1.00 (reference)	434 (66.87)	149 (65.07)	1.00 (reference)	1.00 (reference)
Overweight	252 (19.11)	127 (18.09)	0.92 (0.72~1.17)	0.96 (0.74~1.25)	120 (18.49)	49 (21.40)	1.19 (0.81~1.74)	1.23 (0.81~1.87)
Obesity	34 (2.58)	19 (2.71)	1.02 (0.58~1.81)	1.03 (0.56~1.91)	16 (2.47)	6 (2.62)	1.09 (0.42~2.84)	1.27 (0.45~3.55)
*P* for trend			0.682	0.909			0.366	0.307
**BMI 10 years before interview**								
Underweight	68 (5.16)	70 (9.97)	1.80 (1.26~2.56)	1.60 (1.09~2.34)	40 (6.16)	22 (9.61)	1.57 (0.90~2.73)	1.43 (0.76~2.68)
Normal	785 (59.51)	449 (63.96)	1.00 (reference)	1.00 (reference)	411 (63.33)	144 (62.88)	1.00 (reference)	1.00 (reference)
Overweight	372 (28.20)	155 (22.08)	0.73 (0.58~0.91)	0.76 (0.60~0.96)	165 (25.42)	55 (24.02)	0.95 (0.66~1.36)	1.01 (0.69~1.49)
Obesity	94 (7.13)	28 (3.99)	0.52 (0.34~0.81)	0.55 (0.34~0.88)	33 (5.08)	8 (3.49)	0.69 (0.31~1.53)	0.57 (0.23~1.45)
*P* for trend			**<0.001**	**<0.001**			0.113	0.198
**Body shape at age 20**								
Shape 1	74 (5.61)	47 (6.70)	1.17 (0.79~1.73)	1.28 (0.84~1.95)	35 (5.39)	6 (2.62)	0.50 (0.20~1.24)	0.51 (0.20~1.33)
Shape 2	295 (22.37)	150 (21.37)	0.93 (0.73~1.19)	0.92 (0.71~1.20)	158 (24.35)	52 (22.71)	0.97 (0.65~1.45)	0.86 (0.55~1.34)
Shape 3	499 (37.83)	272 (38.75)	1.00 (reference)	1.00 (reference)	238 (36.67)	81 (35.37)	1.00 (reference)	1.00 (reference)
Shape 4	332 (25.17)	166 (23.65)	0.92 (0.72~1.16)	0.96 (0.74~1.24)	158 (24.35)	61 (26.64)	1.13 (0.77~1.67)	1.01 (0.66~1.54)
Shape 5	92 (6.97)	51 (7.26)	1.02 (0.70~1.48)	1.15 (0.77~1.72)	50 (7.70)	23 (10.04)	1.35 (0.78~2.35)	1.66 (0.90~3.05)
Shape 6/7	27 (2.05)	16 (2.28)	1.09 (0.58~2.05)	1.13 (0.57~2.22)	10 (1.54)	6 (2.62)	1.76 (0.62~5.00)	1.76 (0.58~5.36)
*P* for trend			0.779	0.929			**0.036**	**0.018**
**Body shape 10 years before interview**								
Shape 1	34 (2.58)	57 (8.12)	3.03 (1.93~4.77)	3.25 (2.01~5.26)	16 (2.47)	12 (5.24)	2.46 (1.10~5.46)	2.62 (1.10~6.25)
Shape 2	198 (15.01)	136 (19.37)	1.24 (0.95~1.62)	1.23 (0.92~1.64)	117 (18.03)	48 (20.96)	1.35 (0.79~2.07)	1.27 (0.79~2.03)
Shape 3	458 (34.72)	253 (36.04)	1.00 (reference)	1.00 (reference)	230 (35.44)	70 (30.57)	1.00 (reference)	1.00 (reference)
Shape 4	391 (29.64)	156 (22.22)	0.72 (0.57~0.92)	0.80 (0.62~1.04)	189 (29.12)	57 (24.89)	0.99 (0.69~1.48)	1.07 (0.69~1.66)
Shape 5	170 (12.89)	81 (11.54)	0.86 (0.64~1.17)	1.00 (0.72~1.39)	72 (11.09)	37 (16.16)	1.69 (1.26~2.72)	2.12 (1.26~3.59)
Shape 6/7	68 (5.16)	19 (2.71)	0.51 (0.30~0.86)	0.57 (0.32~1.01)	25 (3.85)	5 (2.18)	0.66 (0.24~1.78)	0.61 (0.20~1.88)
*P* for trend			**<0.001**	**<0.001**			0.378	0.938

Abbreviations: GC, gastric cancer; BMI, body mass index; ORs, odd ratios; CIs, confidence intervals; *Hp, Helicobacter pylori.* Note: Boldface indicates that *P* for trend < 0.05. ^a^ Adjusted for age, sex, education, marital status, occupation, sum of missing and filled teeth, daily frequency of brushing teeth, tea drinking, smoking, alcohol drinking, *Hp* infection, job intensity, family wealth score and family history of GC among first-degree relatives.

**Table 4:** The ORs and 95% CIs for change of BMI and body shape in association with risk of GC cases, stratified by *Hp* infection

Change in Anthropometric parameters	*Hp* infection (+)				*Hp* infection (-)			
	Controls N (%)	GC N (%)	Unadjusted OR (95% CI)	Adjusted OR (95% CI)^a^	Controls N (%)	GC N (%)	Unadjusted OR (95% CI)	Adjusted OR (95% CI)^a^
***Change in BMI***								
***From age 20 to 10 years before interview***								
**Underweight**								
Underweight	36 (2.73)	28 (3.99)	1.00 (reference)	1.00 (reference)	22 (3.39)	10 (4.37)	1.00 (reference)	1.00 (reference)
Normal/overweight/obesity	103 (7.81)	38 (5.41)	**0.47 (0.26~0.88)**	**0.40 (0.17~0.93)**	57 (8.78)	15 (6.55)	0.58 (0.23~1.48)	0.74 (0.12~4.44)
**Normal**								
Underweight	28 (2.12)	38 (5.41)	**2.26 (1.36~3.74)**	**1.94 (1.13~3.31)**	17 (2.62)	10 (4.37)	1.65 (0.73~3.71)	1.42 (0.55~3.64)
Normal	606 (45.94)	364 (51.85)	1.00 (reference)	1.00 (reference)	311 (47.92)	111 (48.47)	1.00 (reference)	1.00 (reference)
Overweight/obesity	260 (19.71)	88 (12.54)	**0.56 (0.43~0.74)**	**0.59 (0.44~0.80)**	106 (16.33)	28 (12.23)	0.74 (0.46~1.18)	0.75 (0.44~1.29)
**Overweight/obesity**								
Underweight/normal	101 (7.66)	59 (8.40)	1.24 (0.82~1.87)	1.24 (0.77~1.99)	55 (8.47)	24 (10.48)	1.14 (0.61~2.15)	1.10 (0.52~2.34)
Overweight/obesity	185 (14.03)	87 (12.39)	1.00 (reference)	1.00 (reference)	81 (12.48)	31 (13.54)	1.00 (reference)	1.00 (reference)
***Change in body shape***								
***From age 20 to 10 years before interview***								
**Shape 1/2**								
Shape 1/2	168 (12.74)	121 (17.24)	1.00 (reference)	1.00 (reference)	90 (13.87)	35 (15.28)	1.00 (reference)	1.00 (reference)
Shape 3/4/5/6/7	201 (15.24)	76 (10.83)	**0.52 (0.37~0.75)**	**0.48 (0.32~0.73)**	103 (15.87)	23 (10.04)	0.57 (0.32~1.04)	1.00 (0.45~2.23)
**Shape 3/4**								
Shape 1/2	60 (4.55)	70 (9.97)	**2.26 (1.56~3.27)**	**1.96 (1.30~2.94)**	41 (6.32)	23 (10.04)	1.68 (0.96~2.94)	2.01 (1.06~3.82)
Shape 3/4	623 (47.23)	322 (45.87)	1.00 (reference)	1.00 (reference)	306 (47.15)	102 (44.54)	1.00 (reference)	1.00 (reference)
Shape 5/6/7	148 (11.22)	46 (6.55)	**0.60 (0.42~0.86)**	0.71 (0.48~1.04)	49 (7.55)	17 (7.42)	1.04 (0.57~1.89)	1.07 (0.54~2.12)
**Shape 5/6/7**								
Shape 1/2/3/4	54 (4.09)	26 (3.70)	0.76 (0.41~1.40)	0.79 (0.38~1.65)	26 (4.01)	11 (4.80)	0.80 (0.32~1.98)	0.58 (0.13~2.62)
Shape 5/6/7	65 (4.93)	41 (5.84)	1.00 (reference)	1.00 (reference)	34 (5.24)	18 (7.86)	1.00 (reference)	1.00 (reference)

Abbreviations: GC, gastric cancer; BMI, body mass index; ORs, odd ratios; CIs, confidence intervals; *Hp, Helicobacter pylori*. Note: Boldface indicates significant associations. ^a^ Adjusted for age, sex, education, marital status, occupation, sum of missing and filled teeth, daily frequency of brushing teeth, tea drinking, smoking, alcohol drinking, *Hp* infection, job intensity, family wealth score and family history of GC among first-degree relatives.

## Abbreviations

GC: gastric cancer
*Hp*: *Helicobacter pylori*
EGJC: esophagogastric-junction cancer
TGC: true gastric cancer
BMI: body mass index
OR: odds ratio
CI: confidence interval
IARC: the International Agency for Research on Cancer
