# Supplementary material for: Changes of Body Mass Index and Body Shape in relation to risk of Gastric Cancer: A population-based case-control study
Source: J Cancer. 2021 Mar 23;12(10):3089–97. doi: 10.7150/jca.56149 (PMC8040898; doi:10.7150/jca.56149)
Supplement: Supplementary file 1 — Supplementary figures and tables. [file jcav12p3089s1.pdf]

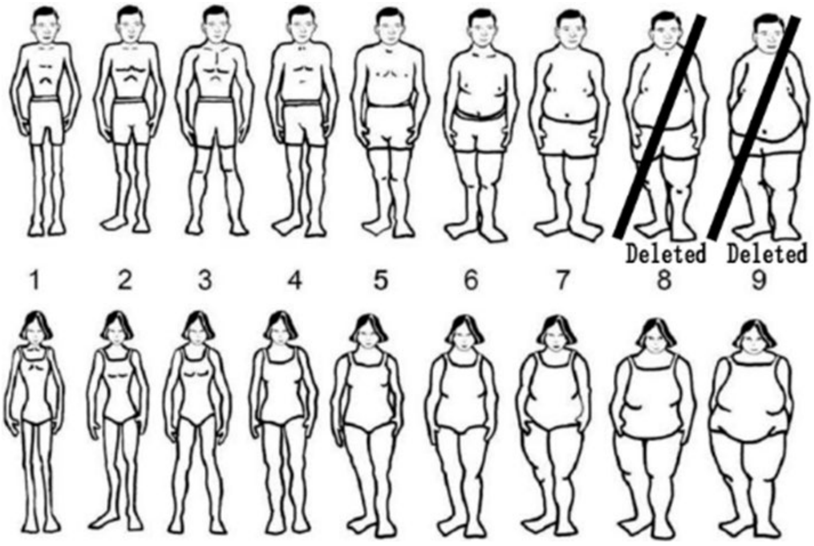

Figure S1. Revised body shape in men (top) and women (bottom).

Table S1. The *OR*s and 95% *CI*s for BMI and body shape in association with risk of EGJC and TGC

**Anthropometric parameters**	**Controls N (%)**	**EGJC N (%)**	**Unadjusted *OR* (95% *CI*)**	**Adjusted *OR* (95% *CI*) [a]**	**TGC N (%)**	**Unadjusted *OR* (95% *CI*)**	**Adjusted *OR* (95% *CI*) [a]**
BMI at age 20							
Underweight	221 (11.11)	41 (9.51)	0.81 (0.57~1.15)	0.90 (0.62~1.31)	51 (10.08)	0.93 (0.67~1.29)	1.00 (0.70~1.41)
Normal	1343 (67.52)	309 (71.69)	1.00 (reference)	1.00 (reference)	332 (65.61)	1.00 (reference)	1.00 (reference)
Overweight	375 (18.85)	73 (16.94)	0.85 (0.64~1.12)	0.87 (0.64~1.16)	106 (20.95)	1.14 (0.89~1.46)	1.18 (0.90~1.54)
Obesity	50 (2.51)	8 (1.86)	0.70 (0.33~1.48)	0.61 (0.27~1.42)	17 (3.36)	1.38 (0.78~2.42)	1.58 (0.87~2.85)
P for trend			0.592	0.371		0.118	0.103
BMI 10 years before interview							
Underweight	110 (5.53)	42 (9.74)	1.64 (1.12~2.40)	1.47 (0.97~2.24)	51 (10.08)	1.79 (1.26~2.55)	1.64 (1.11~2.42)
Normal	1208 (60.73)	281 (65.20)	1.00 (reference)	1.00 (reference)	313 (61.86)	1.00 (reference)	1.00 (reference)
Overweight	542 (27.25)	94 (21.81)	0.75 (0.58~0.96)	0.78 (0.60~1.03)	120 (23.72)	0.85 (0.68~1.08)	0.84 (0.65~1.08)
Obesity	129 (6.49)	14 (3.25)	0.47 (0.26~0.82)	0.44 (0.24~0.83)	22 (4.35)	0.66 (0.41~1.05)	0.63 (0.38~1.04)
P for trend			**<0.001**	**<0.001**		**<0.001**	**0.001**
Body shape at age 20							
Shape 1	110 (5.53)	25 (5.80)	1.10 (0.69~1.75)	1.19 (0.73~1.97)	28 (5.53)	0.94 (0.60~1.47)	1.10 (0.69~1.76)
Shape 2	458 (23.03)	101 (23.43)	1.07 (0.81~1.40)	1.15 (0.86~1.55)	101 (19.96)	0.82 (0.63~1.06)	0.78 (0.58~1.04)
Shape 3	744 (37.41)	154 (35.73)	1.00 (reference)	1.00 (reference)	201 (39.72)	1.00 (reference)	1.00 (reference)

Shape 4	496 (24.94)	111 (25.75)	1.08 (0.83~1.42)	1.11 (0.83~1.49)	119 (23.52)	0.89 (0.69~1.14)	0.90 (0.68~1.18)
Shape 5	143 (7.19)	33 (7.66)	1.11 (0.74~1.69)	1.26 (0.81~1.97)	42 (8.30)	1.09 (0.75~1.59)	1.30 (0.87~1.94)
Shape 6/7	38 (1.91)	7 (1.62)	0.89 (0.39~2.03)	0.85 (0.36~2.03)	15 (2.96)	1.46 (0.79~2.71)	1.61 (0.84~3.09)
P for trend			0.992	0.869		0.191	0.091
Body shape 10 years before interview							
Shape 1	50 (2.51)	37 (8.58)	3.48 (2.19~5.51)	3.48 (2.10~5.78)	32 (6.32)	2.54 (1.58~4.08)	3.02 (1.83~5.00)
Shape 2	318 (15.99)	79 (18.33)	1.17 (0.86~1.58)	1.19 (0.86~1.65)	106 (20.95)	1.32 (1.01~1.74)	1.24 (0.91~1.67)
Shape 3	695 (34.94)	148 (34.34)	1.00 (reference)	1.00 (reference)	175 (34.58)	1.00 (reference)	1.00 (reference)
Shape 4	587 (29.51)	110 (25.52)	0.88 (0.67~1.15)	1.00 (0.75~1.33)	107 (20.95)	0.72 (0.56~0.94)	0.74 (0.56~0.98)
Shape 5	243 (12.22)	48 (11.14)	0.93 (0.65~1.33)	1.01 (0.69~1.49)	71 (14.03)	1.16 (0.85~1.59)	1.27 (0.91~1.76)
Shape 6/7	96 (4.83)	9 (2.09)	0.44 (0.22~0.89)	0.48 (0.22~1.03)	15 (2.96)	0.62 (0.35~1.10)	0.67 (0.36~1.23)
P for trend			**<0.001**	**<0.001**		**<0.001**	**0.001**

Abbreviations: BMI, body mass index; *OR*s, odd ratios; *CI*s, confidence intervals; EGJC, esophagogastric-junction cancer; TGC, true gastric cancer.

Note: Boldface indicates that *P* for trend < 0.05.

[a] Adjusted for age, sex, education, marital status, occupation, sum of missing and filled teeth, daily frequency of brushing teeth, tea drinking, smoking, alcohol drinking, *Hp* infection, job intensity, family wealth score and family history of GC among first-degree relatives.

Table S2. The *OR*s and 95% *CI*s for BMI and body shape in association with risk of intestinal type and diffuse type GC

**Anthropometric parameters**	**Controls N (%)**	**Intestinal type N (%)**	**Unadjusted *OR* (95% *CI*)**	**Adjusted *OR* (95% *CI*) [a]**	**Diffuse type N (%)**	**Unadjusted *OR* (95% *CI*)**	**Adjusted *OR* (95% *CI*) [a]**
BMI at age 20							
Underweight	221 (11.11)	38 (8.33)	0.73 (0.51~1.05)	0.79 (0.54~1.17)	37 (11.01)	1.00 (0.69~1.46)	1.12 (0.75~1.67)
Normal	1343 (67.52)	316 (69.30)	1.00 (reference)	1.00 (reference)	224 (66.67)	1.00 (reference)	1.00 (reference)
Overweight	375 (18.85)	88 (19.30)	1.00 (0.77~1.30)	0.98 (0.74~1.31)	67 (19.94)	1.07 (0.80~1.44)	1.13 (0.82~1.55)
Obesity	50 (2.51)	14 (3.07)	1.19 (0.65~2.18)	1.18 (0.61~2.31)	8 (2.38)	0.96 (0.45~2.05)	1.05 (0.48~2.31)
P for trend			0.177	0.365		0.814	0.826
BMI 10 years before interview							
Underweight	110 (5.53)	48 (10.53)	1.83 (1.27~2.63)	1.63 (1.09~2.43)	30 (8.93)	1.55 (1.01~2.38)	1.42 (0.89~2.27)
Normal	1208 (60.73)	288 (63.16)	1.00 (reference)	1.00 (reference)	213 (63.39)	1.00 (reference)	1.00 (reference)
Overweight	542 (27.25)	103 (22.59)	0.80 (0.62~1.02)	0.82 (0.63~1.07)	82 (24.40)	0.86 (0.65~1.13)	0.83 (0.62~1.11)
Obesity	129 (6.49)	17 (3.73)	0.55 (0.33~0.93)	0.50 (0.28~0.89)	11 (3.27)	0.48 (0.26~0.91)	0.46 (0.23~0.90)
P for trend			**<0.001**	**<0.001**		**0.002**	**0.003**
Body shape at age 20							
Shape 1	110 (5.53)	24 (5.26)	0.89 (0.56~1.43)	1.01 (0.61~1.67)	21 (6.25)	1.26 (0.76~2.09)	1.49 (0.88~2.52)
Shape 2	458 (23.03)	96 (21.05)	0.86 (0.65~1.13)	0.91 (0.68~1.23)	71 (21.13)	1.02 (0.74~1.40)	0.95 (0.67~1.34)
Shape 3	744 (37.41)	182 (39.91)	1.00 (reference)	1.00 (reference)	113 (33.63)	1.00 (reference)	1.00 (reference)
Shape 4	496 (24.94)	106 (23.25)	0.87 (0.67~1.14)	0.91 (0.68~1.21)	94 (27.98)	1.25 (0.93~1.68)	1.30 (0.95~1.78)
Shape 5	143 (7.19)	38 (8.33)	1.09 (0.73~1.61)	1.19 (0.78~1.83)	26 (7.74)	1.20 (0.75~1.90)	1.49 (0.92~2.42)
Shape 6/7	38 (1.91)	10 (2.19)	1.08 (0.53~2.20)	1.12 (0.52~2.40)	11 (3.27)	1.91 (0.95~3.84)	2.00 (0.96~4.16)
P for trend			0.478	0.572		0.174	0.068

Body shape 10 years before interview							
Shape 1	50 (2.51)	30 (6.94)	2.51 (1.55~4.07)	2.61 (1.54~4.43)	25 (7.44)	3.16 (1.88~5.32)	3.74 (2.16~6.48)
Shape 2	318 (15.99)	94 (19.86)	1.24 (0.93~1.65)	1.16 (0.84~1.58)	62 (18.45)	1.23 (0.88~1.73)	1.18 (0.82~1.70)
Shape 3	695 (34.94)	166 (35.89)	1.00 (reference)	1.00 (reference)	110 (32.74)	1.00 (reference)	1.00 (reference)
Shape 4	587 (29.51)	98 (22.01)	0.70 (0.53~0.92)	0.75 (0.56~1.00)	88 (26.19)	0.95 (0.70~1.28)	1.00 (0.73~1.38)
Shape 5	243 (12.22)	56 (12.68)	0.96 (0.69~1.35)	1.00 (0.70~1.44)	44 (13.10)	1.14 (0.78~1.67)	1.28 (0.86~1.90)
Shape 6/7	96 (4.83)	12 (2.63)	0.52 (0.28~0.98)	0.51 (0.26~1.01)	7 (2.08)	0.46 (0.21~1.02)	0.48 (0.20~1.14)
P for trend			**<0.001**	**<0.001**		**0.001**	**0.007**

Abbreviations: BMI, body mass index; *OR*s, odd ratios; *CI*s, confidence intervals; GC, gastric cancer.

Note: Boldface indicates that *P* for trend < 0.05.

[a] Adjusted for age, sex, education, marital status, occupation, sum of missing and filled teeth, daily frequency of brushing teeth, tea drinking, smoking, alcohol drinking, *Hp* infection, job intensity, family wealth score and family history of GC among first-degree relatives.

Table S3. The *OR*s and 95% *CI*s for BMI and body shape in association with risk of GC cases, stratified by sex

**Anthropometric parameters**	**Men**				**Women**			
	**Controls N (%)**	**GC N (%)**	**Unadjusted *OR* (95% *CI*)**	**Adjusted *OR* (95% *CI*) [a]**	**Controls N (%)**	**GC N (%)**	**Unadjusted *OR* (95% *CI*)**	**Adjusted *OR* (95% *CI*) [a]**
BMI at age 20								
Underweight	142 (10.36)	60 (8.94)	0.85 (0.62~1.17)	0.93 (0.66~1.31)	79 (12.78)	32 (12.03)	0.94 (0.60~1.48)	0.96 (0.59~1.56)
Normal	956 (69.73)	475 (70.79)	1.00 (reference)	1.00 (reference)	387 (62.62)	166 (62.41)	1.00 (reference)	1.00 (reference)
Overweight	242 (17.65)	117 (17.44)	0.97 (0.76~1.25)	0.96 (0.73~1.26)	133 (21.52)	62 (23.31)	1.09 (0.76~1.55)	1.17 (0.80~1.71)
Obesity	31 (2.26)	19 (2.83)	1.23 (0.69~2.21)	1.42 (0.75~2.70)	19 (3.07)	6 (2.26)	0.74 (0.29~1.88)	0.74 (0.27~1.98)
P for trend			0.393	0.520			0.889	0.730
BMI 10 years before interview								
Underweight	57 (4.16)	61 (9.09)	2.13 (1.46~3.11)	1.75 (1.15~2.65)	53 (8.58)	32 (12.03)	1.31 (0.81~2.11)	1.30 (0.77~2.20)
Normal	864 (63.02)	435 (64.83)	1.00 (reference)	1.00 (reference)	344 (55.66)	159 (59.77)	1.00 (reference)	1.00 (reference)
Overweight	367 (26.77)	148 (22.06)	0.80 (0.64~1.00)	0.83 (0.66~1.06)	175 (28.32)	66 (24.81)	0.82 (0.58~1.15)	0.77 (0.54~1.12)
Obesity	83 (6.05)	27 (4.02)	0.65 (0.41~1.01)	0.59 (0.36~0.98)	46 (7.44)	9 (3.38)	0.42 (0.20~0.89)	0.45 (0.21~0.98)
P for trend			**<0.001**	**0.001**			**0.004**	**0.007**
Body shape at age 20								
Shape 1	78 (5.69)	32 (4.77)	0.83 (0.54~1.29)	0.91 (0.57~1.45)	32 (5.18)	21 (7.89)	1.50 (0.82~2.74)	1.59 (0.83~3.06)
Shape 2	330 (24.07)	155 (23.10)	0.95 (0.75~1.21)	0.98 (0.75~1.27)	128 (20.71)	47 (17.67)	0.84 (0.55~1.27)	0.81 (0.52~1.28)

Shape 3	536 (39.10)	264 (39.34)	1.00 (reference)	1.00 (reference)	208 (33.66)	91 (34.21)	1.00 (reference)	1.00 (reference)
Shape 4	325 (23.71)	159 (23.70)	0.99 (0.78~1.26)	0.99 (0.76~1.29)	171 (27.67)	71 (26.69)	0.95 (0.66~1.37)	0.98 (0.65~1.48)
Shape 5	78 (5.69)	47 (7.00)	1.22 (0.83~1.81)	1.44 (0.94~2.21)	65 (10.52)	28 (10.53)	0.98 (0.59~1.63)	1.16 (0.67~2.01)
Shape 6/7	24 (1.75)	14 (2.09)	1.18 (0.60~2.33)	1.32 (0.64~2.73)	14 (2.27)	8 (3.01)	1.31 (0.53~3.22)	1.45 (0.55~3.80)
P for trend			0.192	0.170			0.893	0.744
Body shape 10 years before interview								
Shape 1	32 (2.33)	45 (6.71)	3.02 (1.87~4.88)	3.21 (1.91~5.40)	18 (2.91)	24 9.02)	2.87 (1.48~556)	3.61 (1.74~7.49)
Shape 2	217 (15.83)	144 (21.46)	1.43 (1.10~1.85)	1.37 (1.03~1.82)	101 (16.34)	41 (15.41)	0.87 (0.56~1.36)	0.91 (0.56~1.49)
Shape 3	499 (36.40)	232 (34.58)	1.00 (reference)	1.00 (reference)	196 (31.72)	91 (34.21)	1.00 (reference)	1.00 (reference)
Shape 4	410 (29.91)	155 (23.10)	0.81 (0.64~1.04)	0.86 (0.66~1.11)	177 (28.64)	62 (23.31)	0.75 (0.52~1.10)	0.89 (0.59~1.35)
Shape 5	154 (11.23)	79 (11.77)	1.10 (0.81~1.51)	1.24 (0.88~1.74)	89 (14.40)	40 (15.04)	0.97 (0.62~1.52)	1.19 (0.73~1.93)
Shape 6/7	59 (4.30)	16 (2.38)	0.58 (0.33~1.04)	0.63 (0.33~1.20)	37 (5.99)	8 (3.01)	0.47 (0.21~1.04)	0.55 (0.24~1.27)
P for trend			**<0.001**	**<0.001**			**0.006**	**0.041**

Abbreviations: GC, gastric cancer; BMI, body mass index; *OR*s, odd ratios; *CI*s, confidence intervals.

Note: Boldface indicates that *P* for trend < 0.05.

[a] Adjusted for age, sex, education, marital status, occupation, sum of missing and filled teeth, daily frequency of brushing teeth, tea drinking, smoking, alcohol drinking, *Hp* infection, job intensity, family wealth score and family history of GC among first-degree relatives.
